# Serum HPV16 E7 Oncoprotein Is a Recurrence Marker of Oropharyngeal Squamous Cell Carcinomas

**DOI:** 10.3390/cancers13133370

**Published:** 2021-07-05

**Authors:** Lucia Oton-Gonzalez, John Charles Rotondo, Carmen Lanzillotti, Elisa Mazzoni, Ilaria Bononi, Maria Rosa Iaquinta, Luca Cerritelli, Nicola Malagutti, Andrea Ciorba, Chiara Bianchini, Stefano Pelucchi, Mauro Tognon, Fernanda Martini

**Affiliations:** 1 Department of Medical Sciences, University of Ferrara, 44121 Ferrara, Italy; tnglcu@unife.it (L.O.-G.); rtnjnc@unife.it (J.C.R.); carmen.lanzillotti@unife.it (C.L.); elisa.mazzoni@unife.it (E.M.); mariarosa.iaquinta@unife.it (M.R.I.); 2 Department of Translational Medicine and for Romagna, University of Ferrara, 44121 Ferrara, Italy; ilaria.bononi@unife.it; 3 ENT Unit, Department of Neuroscience and Rehabilitation, University Hospital of Ferrara, 44121 Ferrara, Italy; luca.cerritelli@unife.it (L.C.); nicola.malagutti@unife.it (N.M.); andrea.ciorba@unife.it (A.C.); chiara.bianchini@unife.it (C.B.); stefano.pelucchi@unife.it (S.P.)

**Keywords:** human papillomavirus, oropharyngeal squamous cell carcinoma, treatment de-escalation, patient stratification, E7 oncoprotein, HPV DNA, HPV antibodies, ELISA

## Abstract

**Simple Summary:**

Classical markers alone, such as HPV DNA, p16 and HPV mRNA expression, are not enough to stratify HPV-positive head and neck squamous cell carcinoma (HNSCC) patients, but when combined with serological markers, the latter are strong indicators of prognosis in oropharyngeal squamous cell carcinoma (OPSCC) patients. Specifically, HPV16 E7 oncoprotein in serum at the time of diagnosis, correlates with disease recurrence and patient overall survival. To our knowledge, this is the first study to investigate HPV E7 oncoprotein in patient serum. The E7 oncoprotein detection in serum at the time of diagnosis may be useful as a non-invasive procedure for HPV-positive OPSCC patient stratification and follow-up, helping to identify patients at risk for tumor recurrence and metastasis during follow-up, and ultimately, providing a tool for clinicians to identify patients for de-escalation treatment or those to be kept under close surveillance.

**Abstract:**

Despite improved prognosis for many HPV-positive head and neck squamous cell carcinomas (HNSCCs), some cases are still marked by recurrence and metastasis. Our study aimed to identify novel biomarkers for patient stratification. Classical HPV markers: HPV-DNA, p16 and HPV mRNA expression were studied in HNSCC (*n* = 67) and controls (*n* = 58) by qPCR. Subsequently, ELISA tests were used for HPV16 L1 antibody and HPV16 E7 oncoprotein detection in serum at diagnosis and follow-up. All markers were correlated to relapse-free survival (RFS) and overall survival (OS). HPV-DNA was found in HNSCCs (29.85%), HPV16-DNA in 95% of cases, HPV16 E7 mRNA was revealed in 93.75%. p16 was overexpressed in 75% of HPV-positive HNSCC compared to negative samples and controls (*p* < 0.001). Classical markers correlated with improved OS (*p* < 0.05). Serological studies showed similar proportions of HPV16 L1 antibodies in all HNSCCs (*p* > 0.05). Serum E7 oncoprotein was present in 30% HPV-positive patients at diagnosis (*p* > 0.05) and correlated to HNSCC HPV16 E7 mRNA (*p* < 0.01), whereas it was associated to worse RFS and OS, especially for oropharyngeal squamous cell carcinoma (OPSCC) (*p* < 0.01). Detection of circulating HPV16 E7 oncoprotein at diagnosis may be useful for stratifying and monitoring HPV-positive HNSCC patients for worse prognosis, providing clinicians a tool for selecting patients for treatment de-escalation.

## 1. Introduction

Human papillomavirus (HPV)-related head and neck squamous cell carcinomas (HNSCCs) are increasing worldwide [1]. Specifically, HPV-positive oropharyngeal squamous cell carcinomas (OPSCC) have increased over the past few years, with approximately 93,000 new OPSCCs diagnosed per year worldwide [2,3,4]. HPV-positive OPSCCs constitute a biologically distinct group of HNSCCs. Indeed, the American Joint Committee on Cancer, 8th edition, reports improved prognosis and treatment outcome for HPV-positive OPSCC patients compared to HPV-negative cases [5,6].

HPV plays an important role in OPSCC onset [7]. The main transforming activity of HPV relies on oncoproteins E6 and E7, which hamper p53 and pRb tumor suppressor protein activities, respectively. Moreover, HPV E6 and E7 oncoproteins are key players in tumor development, accounting for immune escape, angiogenesis, and the formation of a pro-proliferative microenvironment [8].

Optimizing protocols through targeted therapies and personalized treatments is paramount to increase survival rates for all patients [4]. In this context, several clinical trials, such as PATHOS (NCT02215265) or OPTIMA (NCT02258659), are now in progress [9,10] aiming to determine whether treatment de-intensification could improve quality of life for HPV-positive OPSCC patients whilst maintaining high rates of cure. Indeed, even if HPV-positive OPSCC patients usually respond to treatment de-escalation, 10–25% of HPV-positive patients present recurrences and worse prognosis [9,11,12,13].

Hence, correctly stratifying HPV-positive patients is necessary to select optimized treatment [14]. In an effort to improve stratification, many studies investigate HPV status, p16 overexpression which is the surrogate marker of HPV transformation [6,12], and HPV E6/E7 mRNA expression [15]. However, the current stratification system leads to several pitfalls, i.e., (i) HPV might be present as a transient infection, but not active in tumors [15]; (ii) p16 expression is not always observed in HPV-positive tumors [16]; (iii) HPV mRNA levels could be too low for detection [17,18].

Serological testing has gained interest in the past few years for HPV-positive OPSCC prognostic studies. The immune response of the host has been studied in association with both HPV-positive tumors and patient prognosis [4,19]. Serum IgG antibodies against HPV16 L1 capsid protein can be detected several years before OPSCC onset/presentation, but are also cumulative markers of viral exposure [20,21]. Antibodies to HPV16 E6 and E7 oncoproteins at the time of tumor diagnosis may be useful to predict disease-free survival in HPV-positive OPSCC patients [22,23]. However, routine testing for antibodies against HPV oncoproteins are difficult to perform due to the lack of available commercial kits.

Studies on cervical cancer have shown that detection of HPV E6 and E7 oncoproteins in cervical scrapings may constitute valuable markers for disease progression [24,25]. Moreover, the presence of HPV16 E6 and E7 oncoproteins has been demonstrated by direct ELISA in culture supernatant of HPV-positive cervical cancer cell lines SiHa and CaSki, indicating the release of viral oncoproteins from tumor cells [26].

Therefore, we have hypothesized that HPV E6 and E7 oncoproteins could be present in serum from HPV HNSCC patients, and their serum identification could be useful for prognostic purposes. The presence of HPV E6 and E7 oncoproteins in serum from HPV-associated cancer patients is yet to be investigated. Since new kits for testing serum HPV E7 protein are now commercially available new investigations can be carried out.

The aim of this study was to identify markers for HPV-positive HNSCC patient stratification. To this purpose, classical tumor markers, such as HPV DNA, p16 expression and HPV E7 mRNA were studied in different HNSCC subtypes, including OPSCC. Sera of HNSCC patients were analyzed for HPV16 L1 antibody titers and, for the first time, HPV16 E7 oncoprotein levels at the time of tumor diagnosis and during follow-up at 3, 6, 12 and 24 months. Finally, results were correlated to patient relapse-free survival (RFS) and overall survival (OS) at 24 months.

## 2. Materials and Methods

### 2.1. Patient Samples

HNSCC specimens (*n* = 67) from patients, mean age ± standard deviation [SD] 64.94 ± 10.88 years [y] old, were collected for analyses. Tumor-free tonsillar (TFT) samples (*n* = 58), from non-oncological patients who had undergone tonsillar surgery, 39 ± 15.17 y old, were used as controls. Samples were collected consecutively from 2016 to 2020 at the Ear, Nose and Throat (ENT) Clinic (University Hospital of Ferrara, Ferrara, Italy). Inclusion criteria was the histopathological detection of primary HNSCC in patients 18–95 y old, including hidden or occult SCC with lymph node cervical positive histology. Exclusion criteria were radiotherapy and/or chemotherapy treatment. The 8th edition of AJCC classification was used [5]. Tumor and TFT specimens were collected at the time of surgery. Blood specimens were also collected from HNSCC patients at the time of surgery and during patient follow-up at 3, 6, 12, and 24 months.

### 2.2. Nucleic Acid Extraction

DNA/RNA extractions from HNSCC (*n* = 67) and TFT tissues (*n* = 58) were carried out using the AllPrep DNA/RNA/Protein Extraction Kit (Qiagen, Milan, Italy). After quantification using the NanoDrop 2000 (Thermo Scientific, Milan, Italy), DNA and RNA samples were stored at −80 °C until analyses. DNA suitability for PCR analysis was assessed as before [27,28]. Total mRNA was retro-transcribed using the Improm II (Promega, Fitchburg, WI, USA) reverse transcription system [29].

### 2.3. HPV Analysis

HPV (GenBank: K02718.1) screening was performed by quantitative PCR (qPCR) using the GP5+/GP6+ primer pair (Table S1) [27]. DNA (50 ng) was analyzed in 10 µL reactions consisting of 2× SsoAdvanced Universal SYBR Green Supermix, Bio-Rad (Hercules, CA, USA) and 500 nM of each primer. QPCR analyses were performed in triplicate. Thermal cycling was: 95 °C for 5 min, 40 cycles of 95 °C for 15 s and 60 °C for 30 s. To discriminate between HPV genotypes, a final high-resolution melting (HRM) step was added from 65–95 °C, increasing 0.1 °C every 0.03 s. Recombinant plasmids containing DNA sequences from HPV types 6/11/16/18/31/33/45 were used as positive controls. An HPV-negative genomic DNA sample, and a mock sample, without DNA, were used as negative controls. HPV genotyping was done by comparing sample melting curves to the plasmid controls. Quantification of the viral DNA load was performed in comparison to the standard curve of a plasmid-HPV-type specific [29].

### 2.4. Gene Expression Analysis

QPCR was performed for p16 and HPV16 E7 gene expression analyses. Briefly, 50 ng of cDNA were used in 10 μL reactions using 2× SsoAdvanced Universal SYBR Green Supermix (Bio-Rad). A final concentration of 500 nM of each primer was employed (Table S1). Samples were run in triplicate, along with mock samples used as negative controls. Thermal conditions for HPV E7 and p16 were; 95 °C for 5 min and 40 cycles of 95 °C for 15 s followed by a 60 °C for 30 s. Detection of the housekeeping gene glyceraldehyde 3-phosphate dehydrogenase (GAPDH) was used for normalization of mRNA levels and the fold change was calculated using the 2^−ΔΔCt^ method, as done previously [30,31]. Furthermore, data was normalized against the TFT control group.

### 2.5. Detection of Serum HPV16 L1 Antibodies

Upon collection, blood samples were allowed to clot for 15 min at room temperature and then centrifuged at 1300 g for 15 min. Serum HPV16 L1 IgG antibodies were evaluated in HPV-positive (*n* = 20) and HPV-negative (*n* = 8) HNSCC patients at the time of diagnosis (T0) and during follow-up at 3, 6, 12 and 24 months.

HPV16 L1 IgG antibodies were analyzed with a commercial kit (HPV16 L1, Cusabio, Houston, TX, USA). The test was performed according to the manufacturer’s instructions. The signal intensity was measured as Optical Density (OD) at 450 nm (model Multiskan EX, Thermo Electron Corp., Waltham, MA, USA) [32]. The cutoff value was calculated according to the manufacturer’s instructions; an OD sample/OD negative ratio, equal or greater than 2.1, was considered positive.

### 2.6. Serum E7 Oncoprotein Level Detection

Serum HPV16 E7 oncoprotein levels were evaluated in HPV-positive (*n* = 20) and HPV-negative (*n* = 8) HNSCC patients, using the “HPV16 E7 Oncoprotein ELISA Kit” (Cell Biolabs, San Diego, CA, USA), according to the manufacturer’s instructions. The presence or absence of E7 oncoprotein was determined by considering sample absorbance above or below the cutoff value, respectively, calculated as done previously [33]. The cutoff for HPV16 E7 oncoprotein was 0.75 ng/mL. HPV16 E7 oncoprotein variation during the follow-up was assessed by the ratio between protein amount at time of relapse and at previous time point; ratios > 1 indicated increment of protein prior to relapse, while ratios < 1 indicated decrement.

### 2.7. Statistical Analysis

Statistical analyses were carried out using the GraphPad Prism for Windows (version 8.0, GraphPad, San Diego, CA, USA) [34]. The ANOVA test was used to compare the mean between groups for gene expression analyses. Pearson/Spearman correlation tests were used to correlate viral gene expression and HPV DNA load, and E7 oncogene and p16 expression, respectively, and to assess univariate differences of clinicopathological features according to E7 oncoprotein presence in serum. All parameters were correlated to patient’s relapse-free survival (RFS) and overall survival (OS) at 24 months using the Kaplan-Meier model; statistical significance was estimated using the log-rank test. *p* values of less than 0.05 were considered statistically significant for all analyses.

## 3. Results

### 3.1. HPV DNA Analysis

**Figure 1:**
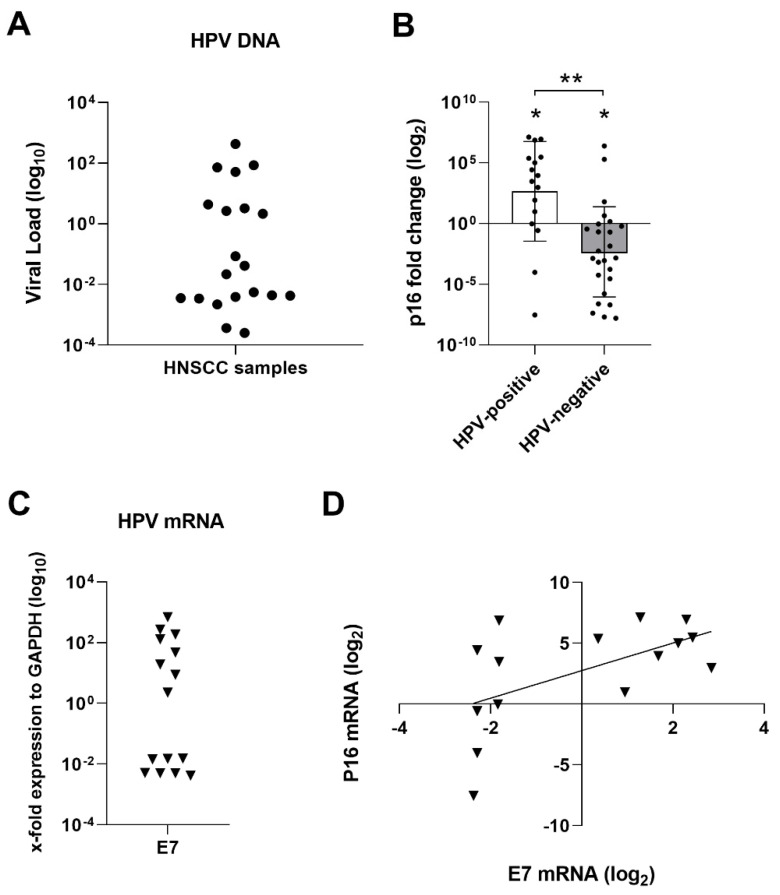
Analysis of classical markers for stratification of HNSCC samples. Statistical significance was indicated as * for *p* < 0.05 and ** for *p* < 0.0001; (**A**) viral load quantification of HPV-positive HNSCC samples by qPCR; (**B**) differential p16 mRNA expression in HNSCC samples analyzed by qPCR; (**C**) Viral E7 mRNA expression in HPV-positive HNSCC samples; (**D**) Spearman correlation analyses between the expression of E7 (log_2_) oncogene and p16 (log_2_) showed correlation (r = 0.59; *p* < 0.05) in HPV-positive HNSCC tumor samples.

HNSCCs and control samples were analyzed for HPV DNA sequences and genotype. HPV DNA was found in 20/67 (29.85%) of HNSCC samples, consisting of 2/20 (10%) oral squamous cell carcinoma (OSCC), 15/20 (75%) OPSCC, 2/20 (10%) hypopharyngeal cancer and 1/20 (5%) laryngeal cancer (Table 1). HPV-genotype was determined by high resolution melting (HRM) to be HPV16 in 19/20 (95%) of the HNSCC HPV-positive cases and HPV33 in 1/20 (5%) of the cases. Control DNAs were found to be HPV11-positive in 1/58 (1.7%) of the cases. Our further studies were hereafter focused on HPV type 16 due to high prevalence in HNSCC. Viral DNA load in cancer specimens ranged from 2.52 × 10^−4^ to 4.26 × 10^2^ copies of HPV DNA per cell (Figure 1A).

### 3.2. p16 Gene Expression

Forty-one out sixty-seven HNSCC matching DNA/RNA samples were available for further analyses. P16 mRNA expression was investigated in HNSCC samples, showing upregulation in 12/16 (75%) of HPV16-positive HNSCC and in 5/25 (20%) of HPV-negative HNSCC samples compared to controls (*p* < 0.001). HPV-positive patients showed overall p16 gene upregulation compared to controls (Mean ± [SD], 2.60 ± 3.98 log_2_ fold, *p* < 0.05) with the exception of two samples that harboured p16 downregulated. HPV-negative samples were downregulated compared to control samples (Mean ± [SD], −2.34 ± 3.71 log_2_ fold, *p* < 0.05). Differences in p16 expression between HPV-positive and -negative were also significant (*p* < 0.0001) (Figure 1B).

### 3.3. HPV mRNA Expression

HPV-positive HNSCC samples were analyzed for HPV16 E7 gene expression by qPCR. Specifically, HPV16 E7 gene expression was analyzed in 16 HPV-positive HNSCC samples. mRNA E7 expression was detected in 15/16 (93.75%) (Figure 1C). Pearson correlation test showed no correlation between the expression levels (log_10_) of E7 and HPV DNA load (r = 0.42, *p* > 0.05). Furthermore, Spearman correlation analyses showed correlation between E7 expression and p16 up-regulation (r = 0.59; *p* < 0.05) (Figure 1D). But, HPV E7 mRNA expression did not correlate to p16 upregulation for all samples, since two samples (one OSCC and one OPSCC) presented E7 expression with p16 downregulation, and one sample presented p16 upregulation but no E7 expression; therefore, p16 is not always a good marker of HPV infection.

### 3.4. Serological Studies

#### 3.4.1. HPV16 L1 Antibody Titer

**Figure 2:**
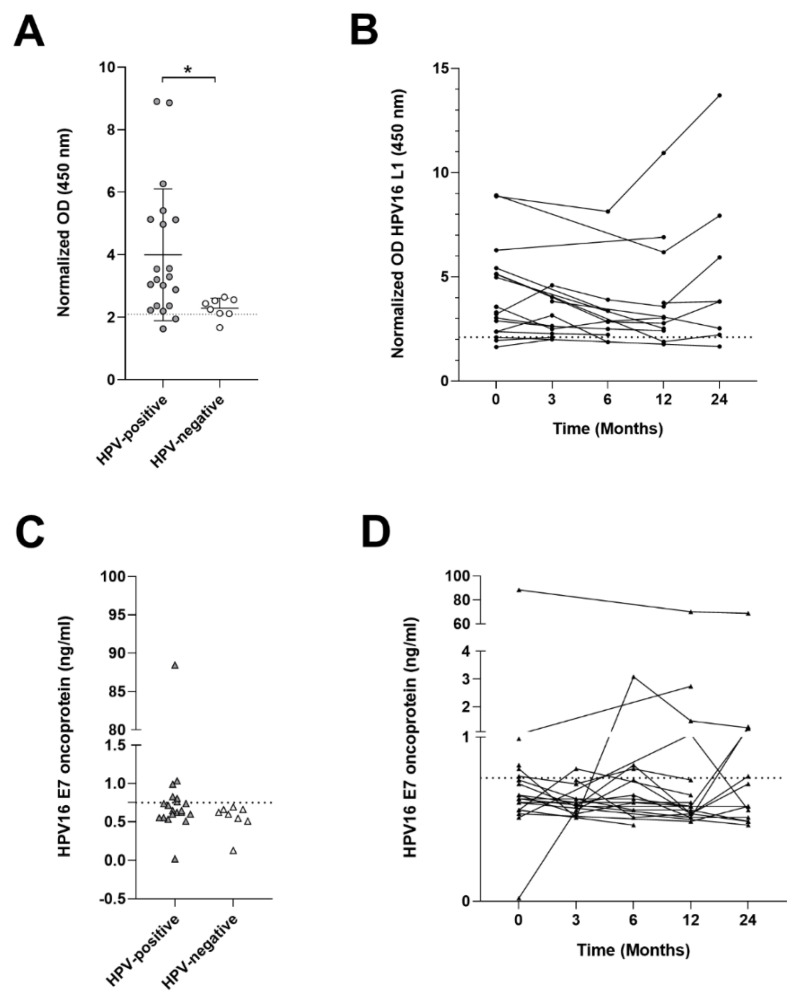
ELISA tests on HNSCC serum samples. Statistical significance was indicated as * for *p* < 0.05; (**A**) Serum antibody levels against HPV16 L1 in HNSCC patients. Differential OD between HPV-positive and HPV-negative patients (*p* < 0.05); (**B**) HPV16 L1 antibody variation during HPV-positive patient follow-up; (**C**) HPV16 E7 oncoprotein quantification in serum shows no difference between HPV-positive and HPV-negative patients (*p* > 0.05); (**D**) HPV16 E7 oncoprotein variation during HPV-positive patient follow-up.

Serum from all HPV-positive (*n* = 20) HNSCC patients (Table 1) and from HPV-negative (*n* = 8) HNSCC patients, consisting of 4/8 (50%) OPSCCs and 4/8 (50%) OSCCs, were tested for HPV16 L1 IgG antibodies. HPV16 L1 antibodies were found with a similar proportion in 18/20 (90%) HPV–positive HNSCC and 7/8 (87.5%) HPV-negative HNSCC patients at T0 (*p* > 0.05). HPV DNA-positive HNSCC patients presented higher Optical Density (OD) readings for antibodies anti-HPV16 L1 compared to HPV-negative (Mean ± [SD], 4.001 ± 2.11 vs. 2.29 ± 0.32; *p* < 0.05) (Figure 2A). Antibody response was further compared during follow-up at 3, 6, 12 and 24 months. Results indicated that HPV16 L1 antibody titers did not vary significantly during follow-up (*p* > 0.05) (Figure 2B).

#### 3.4.2. HPV16 E7 Oncoproteins in Sera

HPV16 E7 oncoprotein (ng/mL) amounts were evaluated at the time of diagnosis and during follow-up at 3, 6, 12 and 24 months. At T0, HPV16 E7 oncoprotein was detected in 6/20 (30%) HPV-positive patient serum and no HPV-negative cases (*p* > 0.05) (Figure 2C). Variation in the amount of E7 oncoprotein during follow-up was studied. Nine out of 20 (45%) patients showed an increment in the amount of oncoprotein during follow-up; 4/9 (44.44%) patients were positive at the time of diagnosis, while 5/9 (55.55%) became positive during follow-up. Two patients out of 20 (10%) positive at the time of diagnosis, presented HPV E7 decrement over-time, and one became negative. HPV16 E7 variation in samples during follow-up resulted statistically insignificant (*p* > 0.05) (Figure 2D). Nine out 20 (45%) patients were E7 negative at T0 and remained negative during follow-up.

Finally, HPV16 E7 oncoprotein levels in serum were studied in correlation to the viral mRNA expression in the tumor samples. Results showed correlation between the amount of HPV16 E7 mRNA expressed in the tumors and E7 oncoprotein in serum (r = 0.79, *p* < 0.01), suggesting that circulating E7 protein may be due to release from the tumor site.

### 3.5. Survival Analysis

#### 3.5.1. RFS and OS in Correlation to HPV DNA, p16 Expression and HPV mRNA

**Figure 3:**
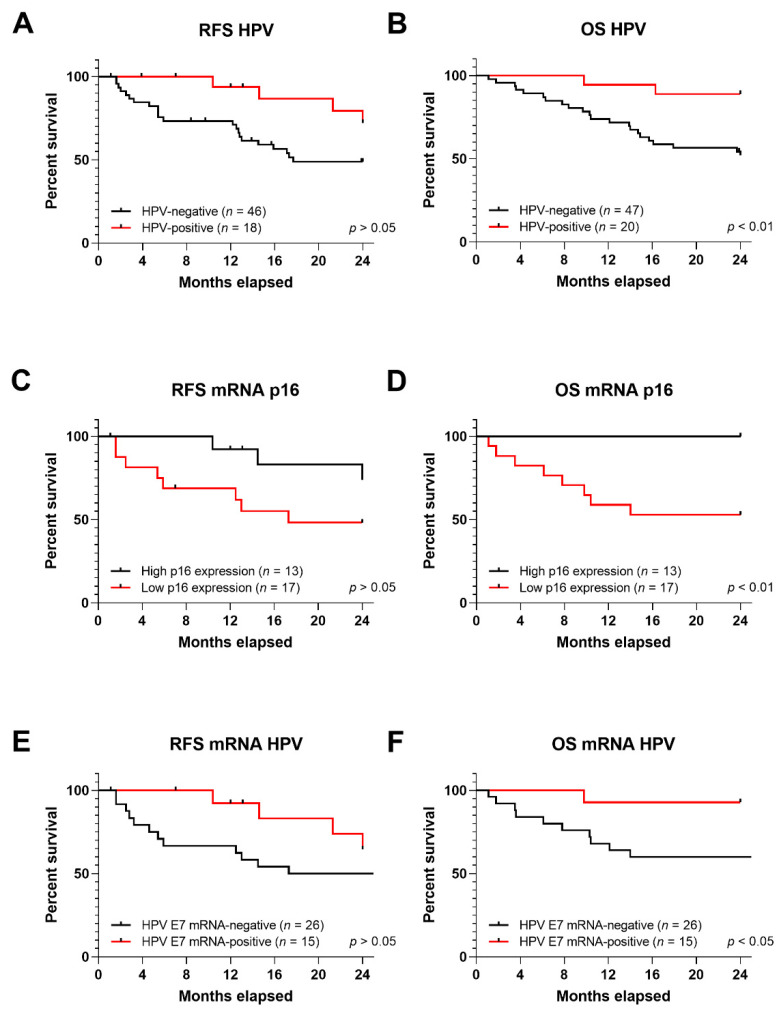
Kaplan-Meier (KM) curves for RFS and OS in HNSCC; KM curves for (**A**) RFS and (**B**) OS for HPV DNA presence in HNSCC tumor samples; KM curves for (**C**) RFS and (**D**) OS for p16 over- or under-expression in HNSCC samples; KM curves for (**E**) RFS and (**F**) OS for HPV E7 mRNA expression in HNSCC tumor samples. Statistical significance was indicated as *p* < 0.01 or *p* < 0.05.

The median follow-up time of this study was 24 months. Relapse free survival (RFS) and overall survival (OS) were assessed in HPV-positive HNSCC patients compared to HPV-negative cases. Different RFS rates were observed; 72.11% and 48.77% for HPV-positive and -negative, respectively (*p* > 0.05) (Figure 3A). Furthermore, OS was improved for HPV-positive patients; 88.89% compared to 52.08% in HPV-negative OPSCCs (*p* < 0.01) (Figure 3B).

To study the effect of p16 expression on survival rate, all HNSCC samples were subdivided into p16-over or –underexpression in the tumor sample. Log_2_ fold change (FC) value (with fixed interval) was used as the cut-off criteria. High and low expression were considered when FC was greater than 1 (*n* = 13) or lower than −1 (*n* = 17), respectively [35]. RFS was 73.84%, in patients carrying p16 upregulation, compared to p16 downregulation, 48.12% (*p* > 0.05) (Figure 3C). OS was 100% in patients with higher p16 expression compared to 52.94% of patients with p16 downregulation (*p* < 0.01) (Figure 3D).

RFS and OS were also assessed for HPV E7 mRNA expression in HNSCCs samples. Samples were divided into expressing E7 oncogene (*n* = 15) and non (*n* = 26). Survival proportions indicated that RFS was 64.61% in patients positive for E7 mRNA, and 48.77% in patients HPV mRNA-negative (*p* > 0.05) (Figure 3E). OS was higher in patients carrying HPV E7 mRNA, 92.85%, compared to HPV mRNA-negative, 52.08% (*p* < 0.05) (Figure 3F).

#### 3.5.2. RFS and OS in Relation to Serum HPV16 L1 Antibodies

**Figure 4:**
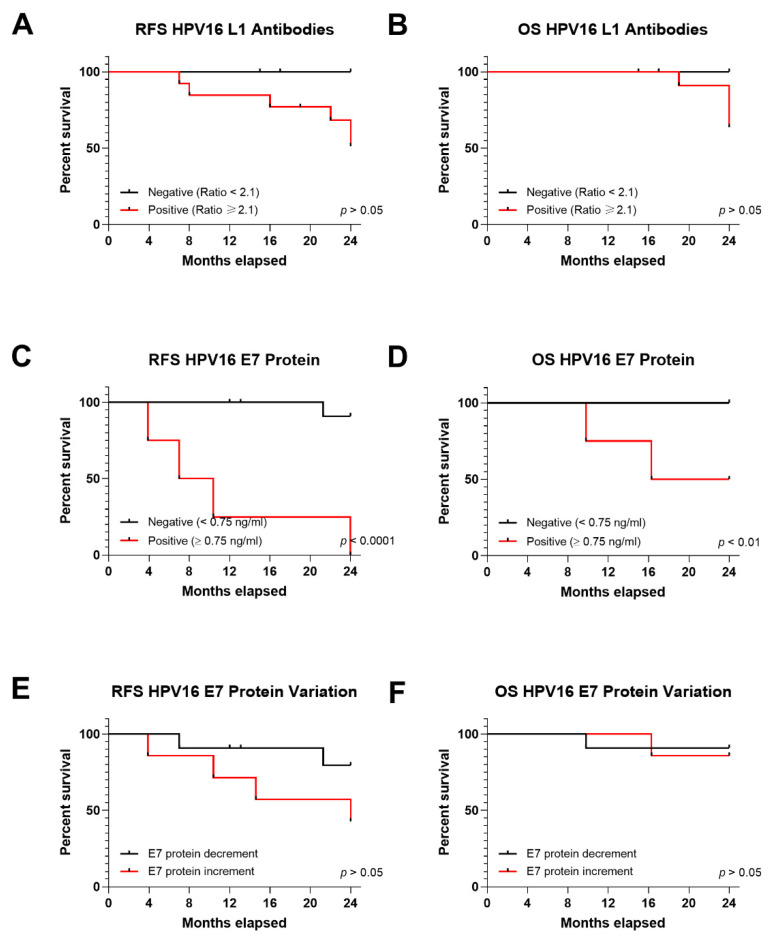
Kaplan-Meier (KM) curves for serological tests representing RFS and OS in HNSCC patients for HPV16 L1 and OPSCC for E7 oncoprotein; KM of (**A**) RFS and (**B**) OS for HPV16 L1 in HNSCC patients; KM of (**C**) RFS and (**D**) OS for HPV E7 oncoprotein in serum from HPV-positive OPSCC patients; KM of (**E**) RFS and (**F**) OS for increment or decrement of E7 oncoprotein in serum from OPSCC patients during follow-up. Statistical significance was indicated as *p* < 0.05.

The next step was to study the association between HPV infection serological markers, such as HPV16 L1 antibody, with patient’s survival. No significant differences were observed for HPV16 L1 antibodies in RFS or OS for HPV-positive patients (*n* = 20) at the time of diagnosis and during follow up. RFS was 51.28% and 100% for HPV16 L1 antibody-positive (*n* = 18), for HPV16 L1 antibody-negative (*n* = 2) patients, respectively (*p* > 0.05) (Figure 4A). OS was also similar between HPV-positive patients and HPV16 L1 antibody positivity or negativity, at 63.64% and 100%, respectively (*p* > 0.05) (Figure 4B). Overall these results indicate that HPV16 L1 is a poor indicator of prognosis and since it is a cumulative marker of exposure, it may be used solely for epidemiological purposes.

#### 3.5.3. RFS and OS in Relation to Serum HPV16 E7 Oncoprotein

HPV16 E7 oncoprotein in serum was correlated to patients’ clinicopathological features (Table 1). Interestingly, E7 oncoprotein in serum was strongly associated to recurrence in HNSCC patients (*p* < 0.0001) and in the OPSCC subgroup (*p* < 0.001). Statistical analyses on other HNSCC subtypes were not possible due to the small sample size (Table 1). RFS was 0% for HNSCC with E7 positivity compared to 90.9% for patients testing negative for E7 protein (*p* < 0.0001) (Figure 4C). OS was 100% and 50% in patients negative and positive for E7 oncoprotein, respectively (*p* < 0.01) (Figure 4D).

The variation in serum E7 oncoprotein was also studied in correlation to patient survival. RFS was 42.85% in HNSCC patients who increased E7 oncoprotein amounts during follow-up, compared to 79.55% in those who experienced E7 decrease (*p* > 0.05) (Figure 4E). OS proportion was 85.71% for patients showing increased E7 oncoprotein, and 90.9% for those showing a decreased E7 oncoprotein (*p* > 0.05) (Figure 4F). These results highlight the importance of patient monitoring for recurrence after circulating HPV E7 oncoprotein being found at the time of diagnosis or increasing levels during follow-up.

### 3.6. TNM Stage in Correlation to OPSCC Patient Prognosis and E7 Oncoprotein in Serum

**Figure 5:**
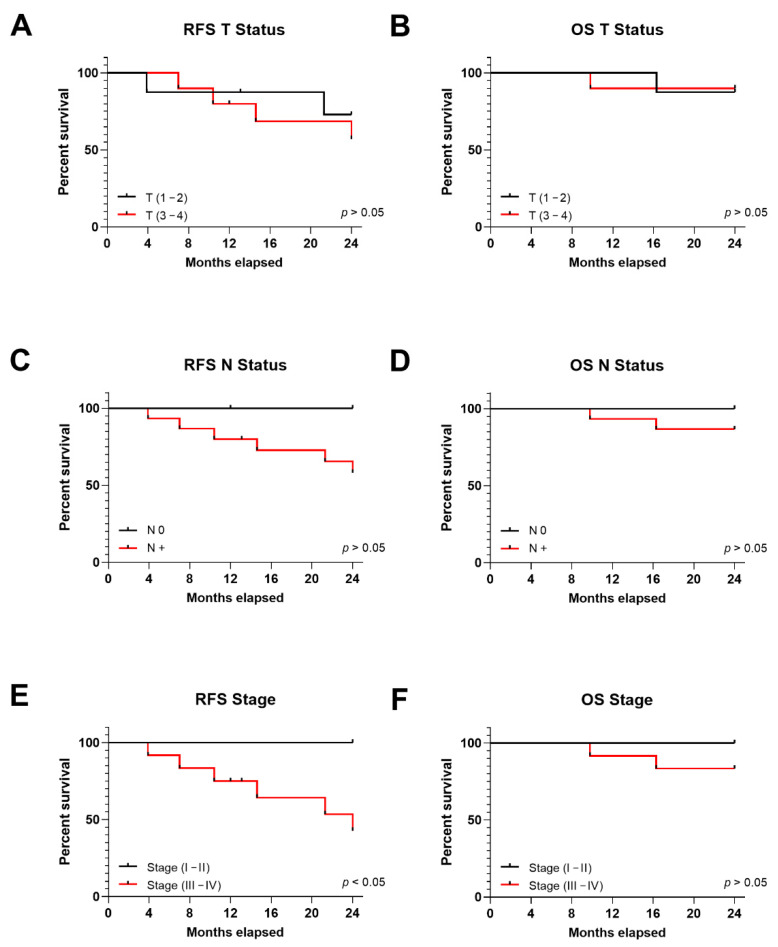
Kaplan-Meier (KM) curves for tumor size (T), node status (N) and stage in HPV-positive HNSCC patients representing RFS and OS; KM representing (**A**) RFS and (**B**) OS for patients divided into tumor size: T (1–2) and T (3–4); KM representing (**C**) RFS and (**D**) OS for patients divided into node status: N0 and N+; KM representing (**E**) RFS and (**F**) OS for patients divided into stages I/II and III/IV; OS for patients divided into stages I/II and III/IV. Statistical significance was indicated as *p* < 0.05.

RFS was 72.9% and 57.14% for patients with T (1–2) and T (3–4), respectively (*p* > 0.05) (Figure 5A), while OS was 87.5% and 90%, respectively (*p* > 0.05) (Figure 5B). Similarly, no statistically significant differences were observed for RFS or OS survival rates when studied in correlation to lymph node involvement; RFS was 100% vs. 58.18% for patients without and with lymph node involvement (*p* > 0.05) (Figure 5C), while OS was similar; 100% and 86.67%, respectively (*p* > 0.05) (Figure 5D).

Patients in stages III/IV are more likely to recur. Indeed, RFS for patients in stage III/IV was 42.86% compared to 100% for patients in stage I/II (*p* < 0.05) (Figure 5E), while OS was similar for both groups 83.33% and 100%, respectively (*p* > 0.05) (Figure 5F).

Lastly, we studied the correlation between serum E7 presence and tumor size, lymph node involvement and disease stage. E7 in serum correlated to tumor size (*p* < 0.05), but not to lymph node involvement in OPSCC (*p* > 0.05) (Table 1). Out of 6 HPV-positive patients with stage I/II, none presented E7 oncoprotein in serum at T0, while 5/12 (41.66%) of the patients in stage III/IV presented E7 oncoprotein in serum (*p* > 0.05).

## 4. Discussion

The current study aims to find markers for recurrence in HPV-positive patients. For patient stratification, we studied classical HPV markers, such as HPV DNA, p16 mRNA and viral mRNA expression. Once stratified, we studied the presence of potential serological markers, i.e., HPV16 L1 antibodies and, for the first time, the HPV E7 oncoprotein. Serological markers were then correlated to patient prognosis.

In an initial screening, we found that 29.85% of HNSCC tumor samples, including 75% OPSCC, harbored HPV-DNA, and 95% tested HPV16-positive. These findings are in accordance to other studies indicating that HPV is found in 25% of all HNSCCs, and in up to 70% of OPSCC tumors [36,37,38,39], whereas 90% of all HPV-positive tumors carried HPV type 16 [40]. P16 mRNA expression was found to be overexpressed in 75% of HPV-positive HNSCC samples. P16 is an established surrogate marker for tumors with transcriptionally active HPV, which is known to be associated with tumors that respond better to therapy and have improved outcomes [41,42]. Yet, not all HPV-positive tumors show p16 gene upregulation, as shown in herein and in previous studies [15]. Transcription of E7 viral oncogene was assessed in HPV-DNA positive patients only, showing 93.75% of HPV16 DNA positive samples expressing the HPV16 E7 oncogene, in agreement with previous studies [30].

Since HPV status has a great impact on patient prognosis for different HNSCCs, such as OPSCC and OSCC [43,44], it is important to stratify patients correctly. In our study, we found that 30% of HPV-positive patients presented recurrence within the first two years of diagnosis, similarly to other studies [9,11,12,13]. Classical HPV infection markers, i.e., HPV DNA, p16 and HPV mRNA showed improved patient OS in our cohort of study, but none of them correlated with recurrence.

Antibody response against L1 was studied for the prevalence of viral infection in HNSCC patients. HPV L1 antibodies are cumulative markers of past and present infection, although their presence does not imply HPV-driven tumorigenesis [45]. Indeed, in our study both HPV-positive and -negative HNSCC patients had antibodies against HPV16 L1 with a similar level of prevalence at 90% and 87.5%, respectively. Interestingly, the antibody titer in HPV-positive patients was higher compared to HPV-negative cases, which could be indicative of active infection.

We also studied the antibody response during the follow-up to monitor disease status, as proposed by Routman et al. [46], but no significant antibody titers change was observed during follow up, indicating that antibody levels against HPV L1 may not be useful in diagnosing or monitoring the disease.

Recently, the study of antibody response against HPV E6/E7 oncoproteins in OPSCC, the major subtype of HNSCC, has been proposed as a marker for disease progression. In spite of good perspectives for both diagnosis and prognosis [19,20,22,23,47], results are still under debate due to the lack of seroconversion in many patients, as was also underlined before for other diseases which are related to viral infections [48]. In a study conducted by Kreimer et al., 57.6% of OPSCC patients remained HPV E6 seronegative during follow-up [47].

To our knowledge, no previous research has been conducted to detect HPV E7 oncoprotein in HNSCC patient serum, while the availability of ELISA kits for oncoprotein detection could rapidly facilitate such study outcomes into clinical use. E7 oncoprotein in serum was specifically found with a frequency of 30% in HPV-positive samples, while the detection of circulating protein at the time of diagnosis strongly correlated to recurrence. Our data are in accordance to other studies showing higher levels of antibodies against HPV oncoproteins at the time of diagnosis in association to a significantly increased risk of recurrence [22,49]. Similar to previous studies on antibody titer variation during follow-up, our investigation found no variation, increment or decrement of E7 oncoprotein in OPSCC serum could be association with patient outcome during the two-year follow-up [20,21,50]. Nevertheless, an increase or decrease in serum E7 during follow-up was observed in patients whether experiencing recurrence or not, respectively, thus, lack of significant correlation between serum E7 level and relapse may be due to limited sample sizes.

It is to be noted that, circulating E7 protein showed correlation with high E7 mRNA expression in the tumor, suggesting that tumor sites may provide the circulating oncoprotein. Circulating E7 protein may be considered a tumorigenesis marker, representing at serological level what occurs at the tumor site. Sources of viral oncoproteins in serum have currently not been established, but some hypotheses could be proposed. Firstly, the transcriptionally active circulating tumor cells may account for the presence of serum viral oncoproteins [13]. Indeed, HPV spreading through blood cells has been previously reported [51], while HPV E6/E7 transcription in circulating tumor cells (CTCs) has been correlated to patient prognosis [13]. Secondly, invasion and the associated development of a tumor vascular bed may result in the release of E6/E7 proteins from the tumor mass, probably as a consequence of necrosis [52]. Thirdly, HPV-positive tumor cells may secrete exosomes containing viral oncoproteins, as has been reported for other DNA viruses [53]. Whatever the mechanism, HPV16 E7 oncoproteins were successfully found in HNSCC patient serum and correlated to patient prognosis. E7 oncoprotein detection in serum at the time of diagnosis displayed strong diagnostic and prognostic reliability in predicting relapses and overall survival in HPV-positive HNSCC patients, especially HPV-positive OPSCC patients. Moreover, since HPV mRNA may be present in HPV-DNA negative samples [54], the analysis of HPV mRNA should be taken into consideration in all HNSCCs to avoid HPV-driven tumors misclassification.

Moreover, E7 oncoprotein also correlated to tumor size, but not lymph node involvement or disease stage. Overall, 41.66% HNSCC patients with high disease stage III/IV presented E7 oncoprotein in serum, while none of those with low stage I/II did so, in agreement with previous serologic studies [20,55], making the detection of E7 oncoprotein in serum an excellent discriminator for HNSCC patients that may relapse, especially for OPSCC patients.

This study demonstrates for the first time, the presence of circulating E7 oncoproteins in serum from HNSCC patients using a direct ELISA assay. Our results indicate that the presence of E7 oncoprotein in OPSCC patient serum at the time of diagnosis is indicative of a higher risk of recurrence. Liquid biopsy for the detection of prognostic markers in HPV-positive OPSCC patients provides valuable information on disease progression and may help stratify and monitor patients over-time; this, can result extremely useful for patients presenting persistent or occult tumors. For future studies, in order to increase the statistical power of the study, a larger sample size for all HNSCC subtypes will be considered. This study takes medicine one step closer to correct patient stratification for therapy de-intensification. The combination of classical markers with serological markers, may be used to plan personalized treatment strategies for HPV-positive patients.

## 5. Conclusions

Detection of circulating HPV E7 oncoprotein at the time of diagnosis, may be used as non-invasive procedure for patient stratification and follow-up, ultimately providing a tool for clinicians to determine which patients would be good candidates for treatment de-escalation or should be kept under close surveillance.

## Figures and Tables

**Table 1:** Clinicopathological features of HNSCC patients.

Clinicopathological Variables	HPV-Negative	HPV-Positive	*p*-Value	Tumor Site (HPV-Positive)							
				Oral	*p*-Value *	Oropharynx	*p*-Value	Hypopharynx	*p*-Value *	Larynx	*p*-Value *
Tumor Site											
Oral	25/47 (53.20%)	2/20 (10%)									
Oropharynx	13/47 (27.66%)	15/20 (75%)									
Hypopharynx	1/47 (2.13%)	2/20 (10%)									
Larynx	6/47 (12.77%)	1/20 (5%)									
Hidden ^1^	2/47 (4.25%)	-									
Tumor Size											
T1	7/47 (14.89%)	3/20 (15%)	0.225	-	NA	2/15 (13.33%)	0.031	1/2 (50%)	NA	-	NA
T2	14/47 (29.79%)	5/20 (25%)		-		4/15 (26.67%)		1/2 (50%)		-	
T3	9/47 (19.15%)	3/20 (15%)		-		3/15 (20.00%)		-		-	
T4	17/47 (36.17%)	9/20 (45%)		2/2 (100%)		6/15 (40.00%)		-		1/1 (100%)	
Node Status											
N0	9/47 (19.15%)	3/20 (15%)	0.108	-	NA	2/15 (13.33%)	0.096	-	NA	1/1 (100%)	NA
N+	38/47 (80.85%)	17/20 (85%)		2/2 (100%)		13/15 (86.67%)		2/2 (100%)		-	
Clinical Stage											
I	1/47 (2.13%)	1/20 (5%)	0.467	-		1/15 (6.67%)	0.336	-	NA	-	NA
II	7/47 (14.89%)	5/20 (25%)		-		5/15 (33.33%)		-		-	
III	7/47 (14.89%)	5/20 (25%)		-		4/15 (26.67%)		1/2 (50%)		-	
IVa	25/47 (53.19%)	9/20 (45%)		2/2 (100%)		5/15 (33.33%)		1/2 (50%)		1/1 (100%)	
Ivb/c	7/47 (14.89%)	-		-		-		-		-	
Recurrence	16/47 (42.55%)	6/20 (30%)	0.0001	1/2 (50%)	NA	4/15 (26.66%)	0.001	1/2 (50%)	NA	0/1 (0%)	NA
Persistance	4/47 (8.51%)	2/20 (10%)				2/15 (13.33%)					
N/A	2/47 (4.25%)	2/20 (10%)		-		2/15 (13.33%)		-		-	
Tobacco consumption											
No	5/47 (10.64%)	2/20 (10%)	0.481	-	NA	2/15 (13.33%)	0.582	-	NA	-	NA
Ex Smoker	13/47 (27.66%)	7/20 (35%)		-		4/15 (26.67%)		2/2 (100%)		1/1 (100%)	
Smoker	25/47 (53.19%)	7/20 (35%)		2/2 (100%)		5/15 (33.33%)		-		-	
N/A	4/47 (8.51%)	4/20 (20%)		-		4/15 (26.67%)		-		-	
Alcohol consumption											
No	10/47 (21.28%)	5/20 (25%)	0.962	-	NA	4/15 (26.67%)	0.725	-	NA	1/1 (100%)	NA
Ex consumer	3/47 (6.38%)	2/20 (10%)		1/2 (50%)		0/15 (0%)		1/2 (50%)		-	
Consumer	27/47 (57.45%)	9/20 (45%)		1/2 (50%)		7/15 (46.67%)		1/2 (50%)		-	
N/A	7/47 (14.89%)	4/20 (20%)		-		4/15 (26.67%)		-		-	
Age	64.04 ± 11.55	67,05 ± 9,05	0.709	63 ± 1.41	NA	65.73 ± 9.15	0.409	73 ± 1.41	NA	83 ± NA	NA
Gender											
Male	32/47 (68.09%)	17/20 (85%)	0.244	2/2 (100%)	NA	12/15 (80%)	0.289	2/2 (100%)	NA	1/1 (100%)	NA
Female	15/47 (31.91%)	3/20 (15%)		-		3/15 (20%)		-		-	

Clinicopathological variables in HNSCC patients both HPV-negative and HPV-positive. *p*-values are referred to correlation between E7 oncoprotein expression in serum and the different variables in HPV-positive HNSCC patients. ^1^ Hidden or occult tumors refer to SCC with lymph node cervical positive histology. *p* values < 0.05 were considered statistically significant. * Too few pairs were available for correlation analysis.

## Data Availability

The data presented in this study are available on request from the corresponding author.

## Supplementary Materials

The following are available online at https://www.mdpi.com/article/10.3390/cancers13133370/s1, Table S1 Validated primer sets used in qPCR to detect and quantify HPV DNA and both, viral and cellular genes.
