# Supplementary material for: Serum HPV16 E7 Oncoprotein Is a Recurrence Marker of Oropharyngeal Squamous Cell Carcinomas
Source: Cancers (Basel). 2021 Jul 5;13(13):3370. doi: 10.3390/cancers13133370 (PMC8268104; doi:10.3390/cancers13133370)
Supplement: Supplementary file 1 [file cancers-13-03370-s001.zip › cancers-1245765-supplementary.pdf]

Supplementary Materials: Serum HPV16 E7 Oncoprotein Is a Recurrence Marker of Oropharyngeal Squamous Cell Carcinomas

Lucia Oton-Gonzalez, John Charles Rotondo, Carmen Lanzillotti, Elisa Mazzoni, Ilaria Bononi, Maria Rosa Iaquinta, Luca Cerritelli, Nicola Malagutti, Andrea Ciorba, Chiara Bianchini, Stefano Pelucchi, Mauro Tognon and Fernanda Martini

Table S1. Validated primer sets used in qPCR to detect and quantify HPV DNA and both, viral and cellular genes.

	Target	Primers Names	Primers Sequence (5′→ 3′)	Amplicon Size (bp)	Annealing Temp. (°C)	Reference
**DNA**						
Viral	HPV L1	GP5+<br>GP6+	TTTGTTACTGTGGTAGATAC<br>GAAAAATAAACTGTAAATCA	139–145	48	[1]
Host	β-Globin	β-Globin F<br>β-Globin R	TGGGTTTCTGATAGGCACTGACT<br>AACAGCATCAGGAGTGGACAGAT	152	56	[2]
**RNA**						
Viral	HPV16 E7	E7 FWD<br>E7 REV	AGGAGGATGAAATAGATGGTCCAG<br>CTTTGTACGCACAACCGAAGC	112	60	[3]
Host	p16	p16 FWD<br>p16 REV	CCAACGCACCGAATAGTTACG<br>GCGCTGCCCATCATCATG	58	60	[4]
	GAPDH	GAPDH F<br>GAPDH R	GAAGGTGAAGGTCGGAGTC<br>GAAGATGGTGATGGGATTTC	226	60	[5]

Supplementary Materials References:

1. Malagutti, N.; Rotondo, J.C.; Cerritelli, L.; Melchiorri, C.; De Mattei, M.; Selvatici, R.; Oton-Gonzalez, L.; Stomeo, F.; Mazzoli, M.; Borin, M.; et al. High Human Papillomavirus DNA loads in Inflammatory Middle Ear Diseases. *Pathog.* **2020**, *9*, 224, doi:10.3390/pathogens9030224.
2. Martone, T.; Gillio-Tos, A.; De Marco, L.; Fiano, V.; Maule, M.M.; Cavalot, A.L.; Garzaro, M.; Merletti, F.; Cortesina, G. Association Between Hypermethylated Tumor and Paired Surgical Margins in Head and Neck Squamous Cell Carcinomas. *Clin. Cancer Res.* **2007**, *13*, 5089–5094, doi:10.1158/1078-0432.ccr-07-0119.
3. Pett, M.R.; Herdman, M.T.; Palmer, R.D.; Yeo, G.S.H.; Shivji, M.K.; Stanley, M.A.; Coleman, N. Selection of cervical keratinocytes containing integrated HPV16 associates with episome loss and an endogenous antiviral response. *Proc. Natl. Acad. Sci.* **2006**, *103*, 3822–3827, doi:10.1073/pnas.0600078103.
4. Marcoux, S.; Le, O.N.L.; Langlois-Pelletier, C.; Laverdière, C.; Hatami, A.; Robaey, P.; Beauséjour, C.M. Expression of the senescence marker p16INK4a in skin biopsies of acute lymphoblastic leukemia survivors: a pilot study. *Radiat. Oncol.* **2013**, *8*, 252, doi:10.1186/1748-717x-8-252.
5. Xiao, Z.; Liu, Q.; Mao, F.; Wu, J.; Lei, T. TNF-α-Induced VEGF and MMP-9 Expression Promotes Hemorrhagic Transformation in Pituitary Adenomas. *Int. J. Mol. Sci.* **2011**, *12*, 4165–4179, doi:10.3390/ijms12064165.
